# Identifying Food Preferences and Malnutrition in Older Adults in Care Homes: Co-Design Study of a Digital Nutrition Assessment Tool

**DOI:** 10.2196/64661

**Published:** 2025-03-03

**Authors:** Jenni Connelly, Kevin Swingler, Nidia Rodriguez-Sanchez, Anna C Whittaker

**Affiliations:** 1 Faculty of Health Sciences and Sport University of Stirling Stirling United Kingdom; 2 Faculty of Natural Sciences University of Stirling Stirling United Kingdom

**Keywords:** ageing, digital technology, dietary measurement, care homes, co-design, dietary intake, food diary

## Abstract

**Background:**

Malnutrition is a challenge among older adults and can result in serious health consequences. However, the dietary intake monitoring needed to identify malnutrition for early intervention is affected by issues such as difficulty remembering or needing a dietitian to interpret the results.

**Objective:**

This study aims to co-design a tool using automated food classification to monitor dietary intake and food preferences, as well as food-related symptoms and mood and hunger ratings, for use in care homes.

**Methods:**

Participants were 2 separate advisory groups and 2 separate sets of prototype testers. The testers for the first prototype were 10 community-dwelling older adults based in the Stirlingshire area in Scotland who noted their feedback on the tool over 2 weeks in a food diary. The second set of testers consisted of 14 individuals (staff: n=8, 57%; and residents: n=6, 43%) based in 4 care homes in Scotland who provided feedback via interview after testing the tool for a minimum of 3 days. In addition, 130 care home staff across the United Kingdom completed the web-based survey on the tool’s needs and potential routes to pay for it; 2 care home managers took part in follow-up interviews. Data were collected through food diaries, a web-based survey, audio recordings and transcriptions of focus groups and interviews, and research notes. Systematic text condensation was used to describe themes across the different types of data.

**Results:**

Key features identified included ratings of hunger, mood, and gastrointestinal symptoms that could be associated with eating specific foods, as well as a traffic light system to indicate risk. Issues included staff time, Wi-Fi connectivity, and the accurate recognition of pureed food and fortified meals. Different models for potential use and commercialization were identified, including peer support among residents to assist those considered less able, staff-only use of the tool, care home–personalized database menus for easy meal photo selection, and targeted monitoring of residents considered to be at the highest risk using the traffic light system.

**Conclusions:**

The tool was deemed useful for monitoring dietary habits and associated symptoms, but necessary design improvements were identified. These should be incorporated before formal evaluation of the tool as an intervention in this setting. Co-design was vital to help make the tool fit for the intended setting and users.

## Introduction

### Background

Diet is an important component of a healthy lifestyle, and it remains one of the most common challenges among older adults in the United Kingdom [1]. Malnutrition is also a prevalent issue in this population. The World Health Organization defines malnutrition as “deficiencies or excesses in nutrient intake, imbalance of essential nutrients or impaired nutrient utilization” [2]. Approximately 10% of those aged ˃65 years are malnourished or are at risk of malnutrition in the United Kingdom [3], a figure that rose to 25% of those aged ˃60 years during the COVID-19 pandemic [4-6]. The prevalence of severe malnutrition is reported to be as high as 45% in those living in residential care homes [7] but may be worse because approximately 70% of malnutrition in the United Kingdom is unrecognized [8,9]. Malnutrition is strongly associated with frailty, as well as functional and health decline [10-12]; thus, it is important to monitor dietary intake and identify malnutrition risk before symptoms are already evident. However, tracking and understanding the impact of dietary intake is not easy. It is possible for people to become malnourished if they do not eat enough nutritionally rich foods for 2 to 5 days [6,13].

Malnutrition in older adults is commonly identified via the Malnutrition Universal Screening Tool (MUST) [14], and when individuals are classed as medium to high risk, it is recommended to document food and drink intake for 3 days. However, there are difficulties with self-reported food diaries, which can be inaccurate due to recording error, misremembering, and socially desirable responses. Additional barriers include limited staff time in care homes and the need for analysis and interpretation by a dietitian [15].

Computer-based tailored interventions may offer a solution because there is evidence that they have positively impacted dietary monitoring in the general population [16]. Automated food classification is a rapidly advancing field of artificial intelligence research that uses a computer source to identify different foods from an image [17,18]. Creating new nutrition databases or using or adapting existing databases means that it is possible to infer detailed nutritional data from photos of food [19]. Convolutional neural network algorithms are used to identify food automatically from photos, and these are linked to a nutritional database to calculate the calories in the identified foods. Machine learning techniques can then be applied to correlate dietary intake with important individual differences such as food preferences, physical symptoms, and malnutrition risk [20]. Automated food classification can consider the portion size and regularity of food intake, which is crucial to monitor in older people because this can significantly influence malnutrition risk. Linking food intake to preferences and symptoms through technology and artificial intelligence means this information could be used to identify potential risks quickly and accurately. This would provide care staff with information to help monitor older people’s diet and decrease malnutrition risk. Studies that have evaluated photo-based dietary assessment tools have found measurement errors comparable to traditional methods [21], and those that have found it a valid method have focused on a younger population, reporting positive feedback [22,23].

### Objectives

Consequently, following the 6 steps for quality intervention development (6SQuID) [24], we sought to co-design a prototype tool with key stakeholders, which we refer to as a “digital nutritional assessment tool.” We aimed to work with advisory groups (AGs) and participants to develop the key features of a prototype tool for monitoring dietary intake, map this onto food preferences and symptoms, and test the prototype with stakeholders. It is important to note that this study reports on developmental research to identify the tool’s suitability, features needed, and usability, rather than its efficacy at capturing and estimating nutrient intake, which is part of the next stage of development and testing. Its key objectives were to complete the first two 6SQuID steps: (1) defining and understanding the problem and its causes and (2) identifying which causal or contextual factors are modifiable—which have the greatest scope for change and who would benefit the most—using a co-design and coproduction approach. This inclusive approach was taken due to the wish for the resulting tool to have real-life application and translation outside of the academic setting while acknowledging the importance of specifying up front the motivations of coproduction, what outcomes are required for whom, and how these might be achieved [25]. This qualitative approach to data collection is necessarily reflective and conscious and has attempted to follow recently produced resources as a guide to quality coproduction methods in health research [26,27]. In this way, it positions end users as essential to the research process; thus, it is collaborative and equitable [27]. In this study, stakeholders were identified as older adults, the tool software designers, dietitians, and ultimately care home staff and residents themselves.

## Methods

### Recruitment

Participants consisted of 2 AGs and 2 separate sets of prototype testers. Both AGs comprised different older adults and dietitians. Care home staff as well as app and tool designers were consistent across the 2 groups. The testers for the first prototype were community-dwelling older adults and care home employees recruited across Stirlingshire in Scotland. For care home testing, the aim was to recruit 3 care homes, 2 staff members, and 4 to 5 residents in each care home. Care home staff and managers were recruited via social media and Scottish Care newsletters to complete a survey and semistructured interviews, respectively.

### Study Design

The study used a co-design and coproduction approach [27] to determine key components of the tool to be developed and used through multiple iterations of usability feedback from older adults, care staff, and dietitians. The tool was developed using the information provided by the AGs in a collaboration between the research team and the app developers, Game Doctor. The development process involved AGs with key stakeholders in the design process (phase 1); community testing and feedback (phase 2); and care setting feasibility and usability testing, as well as consultation and data gathering with a wider group of care home staff and managers (phase 3). This was a continual process, with feedback resulting in a new build of the tool prototype, to 3 builds. A summary of the timeline for the study design and the different groups involved is shown below in Table 1.

**Table 1:** Timeline and summary of the study design.

	Phase 1				Phase 2								Phase 3				
	Month 1	2	3	4		5	6	7	8	9	10	11		12	13	14	15

AG^a^ 1		✓		✓													
Testing				✓					✓					✓			
Survey										✓	✓					✓	
Interviews											✓				✓		
AG 2												✓					✓

^a^AG: advisory group.

### Procedure

#### Phase 1: AG Recruitment and Co-Design of Tool

According to the 6SQuID framework [24], the first step of intervention development is “defining and understanding the problem.” This was achieved through the creation of the first AG (AG 1) with key stakeholders to develop ideas and identify features that would be vital for a food recognition tool in the care home setting. The study and AG role were advertised through email to the Stirling 1000 Elders email list used by ACW to recruit 2 older adults. A snowball technique was then used to recruit key stakeholders working within nutrition and care settings. Through this process, AG 1 with 6 key stakeholders (older adults: n=2, 33%; care home staff: n=1, 17%; app developers: n=2, 33%; and dietitian: n=1, 17%) was formed. The first session was run face-to-face and was used to define and understand the problem (ie, the need for a food recognition model for dietary monitoring and how artificial intelligence could be used as a potential solution). In addition, AG 1 members were asked to brainstorm key features that the tool should include, such as the identification of symptoms related to eating a particular food and potential rating scales to assess mental and physical well-being after eating. AG 1 also identified potential issues and challenges that would be faced in using the tool with older adults in a health care setting generally.

Feedback from this first AG 1 meeting provided the app developers, Game Doctor, with information on which to base the initial build of the tool and the 2 subsequent builds based on later AG meetings. The LogMeal (AIGecko Technologies SL, Artificial Intelligence and Deep Learning Food Division) photo database used by the tool to identify the foods photographed was selected in collaboration between the research team and app developers based on a review of potential existing services, considering the range of foods recognized and financial costs. The first prototype was then developed by Game Doctor, linked to the photo database, and demonstrated in a second meeting of AG 1 for prototype testing and feedback before bug fixing and independent feasibility testing.

#### Phase 2: Feasibility Testing of the Tool and Food Diaries

This phase was based on 6SQuID step 2: “identify which causal or contextual factors are modifiable—which have the greatest scope for change and who would benefit the most.” We recruited 10 community-dwelling older adults via email to Stirling 1000 Elders. Eligibility included owning an Android mobile phone or tablet capable of downloading the tool. The older adults were provided with a pseudonymized log-in ID for the tool and asked to use it to record each meal over 2 weeks by photographing their plate before and after eating. They were also asked to select the foods present that were identified by the tool or to correct any misidentified foods by entering what the foods circled in the tool were. They could then also rate on a 5-point emoji-based Likert scale their mood, level of hunger after eating, and the presence of up to 7 further symptoms. The symptoms included in the tool were based on feedback from AG 1 and included tiredness, diarrhea, cramping, swallowing difficulties, constipation or discomfort, and bloating. A written diary to complete alongside using the tool was provided to participants to indicate the contents of each meal and note down any problems with using the tool and provide general feedback about it. A third and final meeting of AG 1 was planned to discuss the findings from prototype testing and give the members the opportunity for further testing to provide feedback to the app developer so that a third version of the prototype could be developed. Due to difficulties in arranging a final meeting, AG 1 members were asked to use the tool for a week and then complete a short web-based survey. The survey asked about missing tool features and symptoms, strategies to support the tool’s integration in care settings, and other populations that would benefit from using the tool. This was then used as the basis for discussions among members of the second AG (AG 2).

#### Phase 3

##### Overview

As the tool was intended for eventual use in care homes, phases 1 and 2 were repeated with AG 2, a newly recruited group of 4 key stakeholders (older adults: n=2, 50%; care home staff: n=1, 25%; and dietitian [also an older adult]: n=1, 25%). In addition, a new cohort of tester participants, including staff and residents, was recruited through 4 care homes.

At the first AG 2 meeting, the members discussed version 3 of the prototype and the challenges of its application in care homes.

##### Care Home Testing and Focus Groups

For testing, 8 care homes were contacted through existing networks with information on the study and what was expected of staff and residents. The lead researcher (JC) prepared an introduction to the tool and led a separate discussion at each care home on key features that care home staff and residents wanted to see in this type of tool. Each care home then had access to version 3 of the prototype for 2 weeks, and staff and residents were asked to record food intake and food-related symptoms. The care homes were given an Android tablet preloaded with the tool that could be used to take photos of the food. During this period, residents (n=6) were asked to take photos of all foods they consumed, while staff members (n=8) recorded food intake for other residents, across a minimum of 3 days, ensuring that each type of meal (breakfast, lunch, dinner, and snacks) was recorded at least once. The photos were then added to a timeline, and participants had the option to indicate how hungry they felt after the meal using an emoji-based Likert scale and click on any symptoms they were experiencing from a list of common symptoms, which were then added to the timeline. Participants also entered any verbal preferences and indicated whether they needed support with eating.

After prototype testing, JC conducted semistructured interviews with 5 participants (care home staff: n=4, 80%; and resident: n=1, 20%) to obtain their feedback on the usability and acceptability of the tool. Questions covered topics such as what they liked about the tool, difficulties encountered with its use, missing features (eg, physical or mental symptoms that should be included), additional features that should be included, and advice on the usability of the tool in practice (eg, who would take the photos and whether there was enough time to use the tool during a typical care home day). A second and final AG 2 meeting was convened to talk through the feedback from care homes and suggest future directions.

##### Care Home Staff Perspectives Survey and Manager Interviews

Concurrently with the care home testing, a web-based survey was devised for care home staff that included 16 questions under three headings: (1) “About you” (including job title, organization, and time spent in the care sector), (2) “Nutrition” (including questions about monitoring diet and dietary outcomes in their care home organization), and (3) “Routes to integrate tool.” Of the 16 questions, 6 (38%) were open ended. These were included (under the appropriate headings listed previously) to explore any care home–specific strategies or features that would improve the integration of the tool. The survey was designed to take no longer than 20 minutes to complete. This included a 7-minute video on the web showcasing the tool and its current features, which was required to be watched before completing the survey.

A total of 130 participants (care home staff) were recruited across Scotland via social media (ie, Twitter [subsequently rebranded as X] and LinkedIn), emails to existing partner care homes, and through Scottish Care. The inclusion criteria included any worker from within a UK care home. The survey was available through the advertising link, and, when clicked, the individual was taken to the participant information sheet.

After the survey, semistructured interviews were conducted with care home managers to explore the survey feedback and assess how the tool would fit within their care homes. Care home managers could sign up for these interviews via the last question in the survey.

#### Data Analysis

This project used systematic text condensation [28] across the different types of qualitative data collected. This is a descriptive and explorative method for gathering themes across different types of qualitative data, including interviews, observations, and written text analysis. As such, it represents a pragmatic approach that can turn total impressions of the overall chaos of the data into themes with units of meaning that can then be condensed and synthesized into descriptions and concepts.

#### Ethics Approval

This study was conducted in accordance with the Declaration of Helsinki and approved by the General University Ethics Committee of the University of Stirling (8857; September 7, 2023) for studies involving human participants. As a token of thanks, the participants testing the app received £50 (US $63.32) at the end, once the device had been returned. The feedback provided on the tool was anonymized in terms of users but not per care home. This was carried out in this manner to ensure feedback could be related to the size and location of the care home. Those who took part in the advisory group received £50 (US $63.32) per meeting they attended. For both the survey and the follow-up interviews, participants implied consent by clicking the provided link to take part in the survey.

## Results

### Participants

Participants consisted of 2 separate AGs (AG 1 and AG 2) and 2 separate sets of prototype testers. The testers for the first prototype were 10 community-dwelling older adults and care home employees based in the Stirlingshire area. Four care homes responded about participating in the study. The care home testers consisted of 14 individuals (staff: n=8, 57%; and residents: n=6, 43%) in 4 care homes across Crieff, Stirling, and Fife, differing in size and type of organization (nursing care, older adults only, mixed needs, and dementia specialty or general care). Altogether, 130 individuals completed the web-based survey, with 2 managers volunteering to take part in follow-up interviews.

### Phase 1: AG Recruitment and Co-Design of Tool

AG 1 members met with the research team for 2 hours on 2 occasions over the course of 1 year to design, test, and improve the first protype. They also tested the final prototype and gave feedback before testing in community-dwelling older adults.

Game Doctor staff took part in all AGs and produced an initial prototype in March 2022. Game Doctor. They suggested that the 3-sprint model would work well for the development of up to 3 builds of the initial prototype within the project timeline and budget. At the first meeting of AG 1, key features for the prototype were discussed, and priority was assessed. The need for a food recognition model and key features it should have, such as simple and quick food recognition, were noted. How it would work in the care home environment, issues of data security and consent, symptoms that needed to be included, potential for a mood scale, and overall usability of the tool were also considered. Priorities were summarized into a set of features to be incorporated (Table 2).

**Table 2:** Key features identified through the advisory group, along with the priority level determined in collaboration with Game Doctor.

Feature	ID	Description	Priority
Food tracking via image recognition model	F1V1	Food tracking using off the shelf image recognition model	High
Patient profiles	F2V1	A way to create profiles for each patient or resident using the application	High
Correct food button input	F3V1	Input button to confirm that the model has recognized food accurately	High
Before or after plate recognition	F4V1	Ability of model or application to recognize plates as before or after meal	Medium
Manual input form	F5V1	Manual input form to record symptoms and mood via a 5-point Likert scale	High
Analytics	F6V1	Analytics to track user use of prototype	Medium
Opt-in or consent form	F7V1	Consent form for users during testing of prototype	High
Resident data storage	F8V1	Centralized storage of resident data so multiple devices can access resident data	High
Push notifications	F9V1	Notifications or prompts such as push notifications to attract the user back into the application	Medium

Next, Game Doctor produced a short video to guide participants on how to download the tool and use its initial features (ie, taking photos of plates of food before and after meals, logging symptoms via tick-box selections, and recording mood using a face emoji scale). The basic initial prototype was presented at the second meeting of AG 1. Game Doctor provided a walk-through of the basic prototype and presented different food recognition models Game Doctor. LogMeal was chosen as the food recognition model provider due to the size of its database and the accuracy of food recognition (refer to Table 3 for the models compared). This meeting lasted twice as long as the first session to allow for Game Doctor to talk through the functions of the tool and food recognition model, for the group to try it and test with singular foods, and then test with mixed foods over a restaurant meal. The prototype was tested by taking photos of fruits and vegetables after Game Doctor’s presentation. Food in different presentations and textures was provided on plates for testing with the tool. Basic feedback was given when the group tested the tool with whole meals during dinner, with participants providing feedback on its usability. Other features identified to enhance the tool included having an “after” photo to calculate food eaten; adding an element to note whether food had been fortified (eg, high fat or additional protein added); including additional relevant medical conditions; and keeping the tool simple by, for example, using a tick-box approach and having symptom recording options appear when the “after” photo is uploaded.

**Table 3:** Different food recognition models available that could be integrated into the tool.

Provider	Pricing
Clarifai	Free for academic projects
LogMeal	Depends on project
FoodAI	To be confirmed
BiteAI	To be confirmed
Calorie Mama	To be confirmed

Key design issues noted in feedback included the positioning of the “confirm” screen, which sometimes overlapped the photo taken; how to enter a food if the database does not recognize it and provide a correct option; whether the highlighting circles link to each food on the plate; how to cancel the recognition system if there is an error in recognizing a food; and how plate size would be distinguished to calculate nutrition from different portion sizes. Important features discussed as being needed in the next build, based on feedback from using the tool, included (1) the ability to select resident ID and room number; (2) “before” and “after” photos; (3) adding a food fortification button to indicate the addition of butter, cream, protein powder, and so on; (4) having tick boxes for various symptoms that can be personalized to residents and structuring the page to follow the “after” photo or providing an option to enter symptoms later; (5) plate size option; (6) the option to add medical conditions to the user profile; and (7) having the symptoms page appear right after the second photo, including options to indicate fullness, mood, swallowing difficulties, and so on.

### Phase 2: Feasibility Testing of the Tool and Food Diaries

The next phase of the project involved further testing and feedback by users in their home settings. We recruited 10 community-dwelling older adults to test the tool for 2 weeks. Of the 10 participants, 9 (90%) managed to use the tool, while 1 (10%) had difficulty downloading it and withdrew from the study. Of the 9 participants, 8 (89%) used the tool for the full 2 weeks, while 1 (11%) used it for 1 week. Each day, they documented their food intake and any difficulties encountered with the tool in food diaries. In addition, members of AG 1 tested the tool in free-living conditions over 1 week and submitted comments about their experience through a brief web-based survey. The findings from the diaries and survey covered 3 topics: symptoms, features, and populations. Regarding symptoms, it was noted that the tool monitored a good range of symptoms specific to the target group; however, there were suggestions to include additional symptoms, such as thirst, confusion, and anxiety. Regarding features, it was reported that the tool was easy to use when it connected well with the food recognition model. Most of the group said that they did not feel that any key features were missing, although they noted that testing in care home settings would help identify site-specific needs. Suggested features included an “empty plate” button and a directory of foods. As for other populations and settings that might benefit from the tool, the survey responses highlighted community care, patients with dementia, family carers, and hospitals.

### Phase 3

#### Overview

At the first meeting of AG 2, members suggested that care homes would face unique challenges in using the tool, different from those within the community. These might include potential difficulties in recognizing pureed food, fortified meals, or culturally specific foods (eg, haggis), residents with cognitive impairment not being able to use the tool, and the possibility for staff or visitors to assist with tool use. It was decided that we would trial the tool across a range of care homes with residents of varying abilities to gain direct feedback on these issues.

#### Care Home Testing and Focus Groups

The prototype was tested in 4 care homes with 8 staff members (n=2, 25% in each home) and in 1 care home with 6 residents (older adults: n=4, 67%; and younger adults [aged 54 y and 58 y] with learning disabilities: n=2, 33%). Of the 4 care homes, 3 (75%) did not recruit older adults for the testing due to the cognitive functioning status of their residents who would not be able to use the tool; therefore, the staff members used it themselves. The 6 residents who were recruited all tested the tool themselves. The staff recruited had from 6 months to 20 years of experience of working in the care sector. The older adults recruited were aged between 78 and 84 years and had lived in the care home for at least 6 months. After prototype testing in the care homes, the semistructured interviews with staff (4/8, 50%) and residents (1/6, 17%) identified (1) the pros and cons of using the tool in their care home, (2) how it can be adapted to enhance its usability, and (3) common symptoms reported in the tool. The resident who tested the tool was 1 (50%) of 2 younger residents with learning disabilities (aged 58 y). The main outcomes from these interviews are displayed in Textbox 1.

**Themes emerging from semistructured interviews with care home staff and residents.**

**Key design issues**

- Wi-Fi issues slowed down the uploading of data
- Tool struggled to recognize pureed food
- Camera picked up patterns on plates as food
- Design features are needed to speed up the process

**Key features**

- Staff would prefer photo profiles of residents instead of names
- International Dysphagia Diet Standardization Initiative framework [29] and Malnutrition Universal Screening Tool [14] malnutrition risk score should be built in
- Traffic light system approach to identifying risk
- Self-choose whom to measure option
- Empty plate button
- Monitor fluid intake too
- Pureed food and fortification recognition is essential
- A notification or prompt system as a reminder

**Positive feedback**

- Recognized food well
- Residents liked monitoring their own food intake
- Enjoyed playing around with the tool and learned how to use it quickly

#### Key Design Issues and Usability

All care homes reported difficulties in using the tool when the home Wi-Fi connection was poor:

We had connection issues and found the app to be very slow or would freeze-could there be an easy offline system to use throughout the home that would automatically upload when a strong wifi connection was made?

Staff, care home 2

All care homes reported slow uploads, with the tool freezing; therefore, participants had difficulty clicking on the foods on the screen. This then led to slower identification of foods and a slow search for foods that were not identified, highlighting the need for features that would speed up this process.

A resident who was using the tool revealed that they did not have these issues because they just saved the photo to upload later when their Wi-Fi connection was stronger:

I found the tool easy to use, wifi is terrible here so I just waited until the signal was stronger and uploaded the photo then.

Resident, care home 2

Staff members expressed concern that if they did not upload photos and ratings immediately, they would not be able to do so later due to a change of shift or being given another role or task within the home. Furthermore, choking was identified as a major concern in all participating care homes, and many of the residents were on a pureed diet. The tool struggled to recognize pureed foods, highlighting the need for better pureed food recognition or an alternative solution:

A number of our residents are on pureed diets or we add thickening agents (which contain calories) to some of their foods, the tool could not properly detect it, although funnily enough we use molds shaped like the foods and it could sometimes pick that up.

Staff, care home 1

Staff members also wanted to add a measure of swallowing difficulties to the tool, which would link to food intake and the International Dysphagia Diet Standardization Initiative (IDDSI) framework [25]:

Having a feature and notes in IDDSI, malnutrition risk and fortification is really important for us and our residents.

Staff, care home 4

The current version did not link a swallowing difficulties feature, but adding this would encourage care homes to change the texture of the food.

Finally, when photos were taken of plates, the food recognition function of the tool sometimes misidentified the design patterns on the plate as food:

The plate was empty but it was saying the patterns were food and giving it a value.

Staff, care home 3

However, the more the tool was used, the less this became a problem because users worked out how to delete the circle around the patterned part that was misidentified as food. In addition, continued use improved the tool’s accuracy through machine learning, reducing misidentifications over time.

#### Key Features Missing From the Tool

Regarding features needed in the tool for the care home setting, all homes reported the need for a resident profile instead of the current name function, which takes users straight to the diary:

We need a profile with the resident’s picture which we choose from to quickly find the resident—we have a few residents with the same name.

Staff, care home 2

Staff members wanted to be able to upload a photo of each resident to click on instead of selecting a name, which would take them directly to the resident’s profile. This profile would incorporate relevant information about the resident such as weight, changes in weight, issues with previous foods such as stomach problems or swallowing difficulties, risky foods and allergies, IDDSI [29] and MUST [14] scores, medications that may affect diet, and a category for food monitoring (high risk, medium risk, or low risk). Having the malnutrition risk score assessed via the tool and keeping track of IDDSI scores was a high priority for staff. These features would support staff, particularly new staff who are less familiar with a resident, in ensuring that the correct food is provided:

Choosing the picture would help with our new staff or our bank staff to recognize the resident, we have quite a high turnover here so a photo would quicken the recognition process.

Staff, care home 4

The profile would be accessed, leading to the diary, and a warning signal would be produced when a picture is taken to highlight risky foods for that resident. This was suggested as an important future feature:

If the tool was able to actually warn or prompt staff of certain foods it would really support our staff-we have a lot of residents here who have allergies or are choking hazards so it would support staff unfamiliar with these residents when giving them their meals.

Staff, care home 2

A key concern for care home staff using the tool was the time it would require to take the photo and deliver a meal to the resident while it was still hot. Some homes had 80 residents, and staff would not be able to monitor everyone in addition to their other duties. Upon further discussion, a traffic light system was suggested:

Having something like a traffic light system for overall nutrition risk of the resident as well as risk of the food would be brilliant. We could then monitor our resident over time and give more detailed reports when the dietitian came to who was priority.

Staff, care home 3

This would be a system that would categorize individuals as *green* (does not need regular monitoring), *amber* (needs monitoring but is not of major concern), and *red* (needs monitoring and may need observation or support with eating due to key issues such as swallowing difficulties or choking concerns). Care staff also wanted to be able to choose which residents to monitor because this would help reduce the time spent using the tool by prioritizing residents who needed monitoring the most while eliminating those who did do not require it.

Given concerns over staff time, methods to speed up the tool’s use were highlighted as essential components to integrate. One suggestion was allowing staff to self-select individuals they needed to monitor based on their own experience of who needed support the most:

We know our staff well, we know who has problems when it comes to eating, and we know who to highlight to our manager, if we could choose who to monitor and have the tool to support our statements it would help.

Staff, care home 1

Another suggestion was to add an “empty plate” button:

Being able to just even say they ate everything would really help.

Staff, care home 4

This would eliminate the need for a second photo. A “half-finished” button was also suggested; however, it was highlighted that this could lead to users not knowing what parts of the meal had been eaten, resulting in an inaccurate estimate of nutritional intake:

Being able to click that they ate half would quicken things up, but saying that, we have certain residents who only eat their meat and leave their veggies.

Staff, care home 2

Having a database with preexisting photos based on current care home menus was identified to speed up use of the tool, with the staff only needing to take an “after” photo. Another suggestion to expedite the process was to work with the care homes to integrate their menus into the tool. Of the 4 care homes, 3 (75%) reported a 3-week menu that could be incorporated, and staff would pick from preassigned photos of each meal rather than take “before” photos of all meals. They would then only need to capture “before” photos for individuals with smaller portions or meal modifications (eg, substitution or fortification). Afterward, staff would take “after” photos of unfinished plates, using an “empty plate” button for completely finished meals.

Care staff mentioned the importance of monitoring fluid intake as well as food consumption. Linking fluid intake to bathroom breaks and urinary tract infection diagnoses would motivate staff to provide better fluid care for residents:

If we could monitor what they are drinking and then when we take them to the toilet, particularly during the night, it would be really useful so our staff can monitor who hasn’t drank in a while. It would be even better if the tool could tell us when to stop giving them liquids or if certain liquids made them need more, to prevent them needing at night time.

Staff, care home 3

Staff felt that it would be useful to build a feature into the tool that gave personalized fluid feedback and indicated when best to give out fluids, the best type of fluids to give, and whether there should be any time restrictions to ensure the best level of hydration for each resident.

Given that most residents were on restricted diets—some requiring pureed food due to swallowing difficulties and others needing fortified meals—having these dietary modifications recognized by, or incorporated into, the tool was deemed essential. Staff identified taking multiple photos as the only feasible solution that would still provide accurate nutritional information. This process would involve taking (1) a photo before pureeing, (2) a photo of the pureed meal, and (3) a photo after consumption. At the second meeting of AG 2, it was confirmed that further discussions with app developers would be needed to determine the quickest and most accurate method for implementing this approach.

Residents who tested the tool suggested having a notification or prompt system to remind them to use it. They mentioned that they sometimes forgot to log their food intake and became upset when they missed the opportunity to do so:

I eat at different times to the other residents, and was worried I wasn’t allowed to use it out with mealtimes so I sometimes forgot to take pictures. This was annoying as I really enjoyed using the tool. Will I still have access to it?

Resident, care home 2

Residents reported that being able to set their own prompts or notifications would be beneficial. However, staff did not share this preference because they felt that they themselves would remember to use the tool at mealtimes as part of their work routine. The only instance in which staff considered a prompt system useful was for alerts warning them if a resident’s meal contained an allergen or had previously posed a choking risk.

#### Care Home Staff Survey and Interviews

Altogether, 130 individuals working in the care industry completed the survey. Most of the respondents were health care staff working in a care home (62/130, 47.7%), followed by care workers (25/130, 19.2%), support staff (19/130, 14.6%), and managers (9/130, 6.9%). Of the 9 managers, 2 volunteered to participate in the postsurvey interview. Of the 130 respondents, 60 (46.2%) had worked in the care industry for >5 years, while 30 (23.1%) had worked for <2 years. When asked how they monitored residents’ diets, most (114/130, 87.7%) did not provide specific examples or measurable approaches used in their care homes. Many mentioned menu planning, staff training, and nutritional analysis but without detailing the methods or explaining how food intake was tracked for each resident. Among those who provided examples, methods included written reports (9/16, 56%), diet diaries (4/16, 25%), and computer platforms (2/16, 13%). When asked about additional symptoms that they felt were important to monitor in residents but were not available in the current version of the tool, the most commonly mentioned were hunger (45/130, 34.6%), mood (41/130, 31.5%), and swallowing difficulties (39/130, 30%). However, these symptoms were already included in the tool but may need to be more visually prominent and not limited to being reported only after meals. Other suggested symptoms included tiredness and pain. When asked which features they felt should be added that were not available in the current version, the most common responses were profiles that use photos to identify resident rather than just their name (42/130, 32.3%), a traffic light system (36/130, 27.7%), and integration of IDDSI and MUST scores (36/130, 27.7%). Additional suggestions aligned with feedback from individual care homes, including a regular weigh-in section and fluid intake monitoring. These suggestions were discussed at the second meeting with AG 2, focusing on developing a basic version of the tool that meets general needs across care homes, with the option for individual care homes to add personalized features if deemed essential, provided they were willing to cover the additional cost.

The survey data were supported by the interviews with care home managers. Both managers reported using multiple methods to monitor residents’ diets, although these were not always consistent across staff members or health professionals. They noted that having an app capable of tracking diet, identifying malnutrition, and monitoring associated symptoms would help standardize care across residents and life situations. The managers agreed that such a tool would allow dietary monitoring beyond the care home, including when residents were out with family or hospitalized, ensuring that symptoms could be monitored during these periods. A traffic light system that was visible to staff but not residents was considered highly important to ensure close monitoring of only residents who needed it. The managers were enthused about the tool and using it as part of care but had some concerns. They were worried about the time required to take photos and monitor symptoms, but when prompted during the interview, they came up with strategies themselves to speed up the process. Both managers expressed concerns about the cost of the tool and its uptake. While they acknowledged the tool’s value, they noted that traditional paper methods were more economical unless the tool provided clear value for money. When discussing ways to make this feasible, they suggested (1) making it free for National Health Service (NHS) users, (2) implementing a tiered fee structure for access to different tool features, (3) generating revenue from external users to help subsidize the costs for care homes, and (4) finding funding to support its implementation in care homes.

## Discussion

### Principal Findings

We co-designed a prototype tool that enabled users to take photos of food plates using Android phones or tablets and link foods to a variety of chosen symptoms and mood ratings. The prototype was then tested in care homes. While the ultimate version of the tool would also calculate nutritional intake from the photos, the aim of this study was to co-design the tool to maximize usability. The co-design involved older adults, care home staff, dietitians, app developers, and researchers. The repeated design and test approach ensured that the prototype was co-designed with AGs, with participants’ feedback used to make incremental improvements before testing whether the suggested features were usable and integral to the overall objectives of the tool. This agile methodology implemented by Game Doctor allowed for rapid prototyping and testing of each of the 3 builds by end users. Testing in the community and later in care homes revealed what worked well and what needed updating as well as features that should be included to make the tool specifically suitable for use in care home environments. This collaboration with end users was vital to developing a tool suited for diet and symptom monitoring in care homes.

Care home staff and residents provided feedback on how to ensure that the tool could be implemented in their care home and were very positive about using it overall, although it should be acknowledged that this may have been influenced by the relative experience of the staff. However, the range of experience across the testers gives us confidence that we have captured a range of views. All participants emphasized the tool’s value in supporting staff; facilitating quicker diagnosis of dietary-related symptoms; and enabling earlier detection of malnutrition, which is an important issue in care homes [7]. They reported that they would not need to monitor every resident or use every aspect of the tool but that it would be very valuable to use and could additionally form part of their marketing approach to attract residents.

Care home staff and residents suggested several important features and ways to increase usability. Priority features that will be integrated into future design are enhanced resident profiles with a traffic light system, integration of IDDSI [29] and MUST [14] scores, and recognition of pureed food and fortified meals. Traffic light systems have been found to be successful in dietary monitoring previously by encouraging users to choose lower-calorie alternatives [30]. A similar approach could be applied to identifying “safe foods” in the current targeted group. This would circumvent the need for diet diaries and bringing in a dietitian to interpret data [15], potentially accelerating the recognition of malnutrition with the addition of a nutrition calculator and other tool features to enable appropriate actions to be taken before hospitalization becomes necessary. Previous tools [22,23] that have used photo-based dietary assessment have found this to be a valid, feasible, and user-friendly approach to monitoring dietary intake. However, these tools have focused on younger populations, who may have different dietary requirements and greater familiarity with technology compared to the current target group.

Regarding wider use across care homes and potential methods of commercialization, the care staff survey and manager interviews indicated a preference for the tool to be free for NHS users. This would support its adoption across services and ensure that those who most needed it could fully benefit from its features. Finding funding from the local council or government or having the cost subsidized by nonresident users was brought up as another way to keep costs low or eliminate them for care homes. Managers suggested that even staff members interested in using the tool for their own dietary monitoring could help subsidize costs. When discussing payment for the tool, managers remained positive about using it, provided it was not too expensive, which would divert funding from other activities. Their preference was to pay an annual fee that would give them access to the tool, including updates and maintenance support. A tiered fee structure was also suggested, where costs would increase based on the number of features accessed. Although the managers would prefer the tool to be free, they were still keen to use it even if it was associated with a small fee. The global revenue for nutrition apps was projected to reach US $5.4 billion in 2024, with an expected annual growth rate of 11.2% [31]. This highlights the popularity of dietary monitoring and the importance of working with care home organizations to develop a financially feasible model for the tool.

Co-design between end users, the tool developers, and the research team was integral to this tool’s production. As previous tools [22,23] and reviews of image-assisted dietary assessment methods [32] have focused on younger populations, it was essential to involve older adults in the codevelopment process. The feedback gathered played a crucial role in shaping the tool’s functionality and features, ensuring that it was suited to the intended environment and users. This emphasizes the importance of co-design and coproduction in this type of research and innovation [27]. Applying the 3-sprint approach to design enabled a continual process of refinement, resulting in the production of a tool created by and for older adults and staff in care homes. Without this iterative process, the research team could have estimated which features might be important for users in care homes, but direct lived-experience evidence was essential to verifying whether these were the right features to incorporate and ensuring the tool’s feasibility for the intended end users. However, despite this inclusive approach to ensuring that the tool incorporated the most important features for the setting, several technical improvements are still needed, along with the integration of new features; for example, because this study focused on co-design and feasibility testing, it is not yet clear how well the tool scores in terms of the accuracy of food recognition, and further testing, as well as comparison to similar tools, will be needed to ensure that this critical aspect performs well. Future versions would need to use a specially trained model that is linked to known menus; this should improve food recognition substantially. Other tools have reported mixed accuracy estimates, ranging from 9% to 63% [33]. One advantage of our tool is that it allows manual corrections or direct entry of specific foods and can be directly linked to a care home’s menu plan to increase the accuracy of food recognition. Many similar tools have been criticized for their inability to accurately assess the quantity of food eaten [33]. Our tool addresses this limitation by incorporating “before” and “after” meal photos, enabling it to calculate the actual amount consumed rather than just identifying the food on the plate. Future research is needed to evaluate food quantity estimation as well as food recognition performance. However, the tool has the potential to simplify and speed up malnutrition recognition and dietary monitoring in later life among older adults, who are at higher risk than younger populations.

### Limitations

This study is not without limitations. While the use of co-design and coproduction ensured that the tool included relevant features suited to its intended setting, our testing sample lacked diversity. All care homes recruited were private rather than council funded, and all AG members and tool testers were of White British origin. However, this reflects the typical ethnic composition of the Scottish population [34], and there was diversity in terms of gender, age, occupation, and specific care home site across participants. Future research would benefit from recruiting more diverse samples, including participants from both council-funded and private care homes, as well as recruiting across a range of geographic locations, including inner-city areas where communities are more ethnically diverse. A second limitation was the specific database used, which was developed in Spain and optimized for a more European-style diet. As a result, it did not include many foods commonly consumed in Scotland and misidentified some foods as typical Spanish dishes (eg, paella). However, a future direction for this research is to create a bespoke database of food consumed in Scottish care homes as well as exploring the possibilities of working with individual homes’ menu planners to create a tool specific to each home. This approach could be fundamental to the economic model for maintaining and continually updating the tool as a way of charging for a bespoke service rather than tool *use* in future, allowing us to provide the tool free in other settings, such as the NHS. One limitation of the co-design and coproduction methodology was the diversity of feedback regarding the tool and its usability and key features. While some care homes wanted enhanced features, such as malnutrition risk scores, a traffic light system, and fluid measurement with urinary continence tracking, others preferred to keep the tool’s functions simple to enable ease of use. Further discussions clarified that the enhanced features could be optional, tailored to individual care homes’ needs, and potentially cost associated, which eliminated the contradictions in the feedback.

### Future Directions

Building on the outlined future directions, the next steps for this research project are to incorporate the care home feedback into the next build of the tool to ensure its suitability for care homes. To do this, we plan to apply steps 3 to 6 of the 6SQuID framework [24], test the tool in a more diverse sample of care homes, link it to a specially developed database of food photos reflecting the typical Scottish care home diet, and explore cost-effectiveness options and sustainability strategies to ensure that the tool remains up to date.

### Conclusions

This journey through the co-design of a digital food recognition tool has revealed both its considerable potential and areas for further design improvement and feature enhancement. As we progress toward further prototype refinement and wider testing in diverse care home settings, the feedback and recommendations from this project will guide our approach, ensuring that the tool is practical, feasible, and robustly designed to comprehensively monitor diet and identify malnutrition risk and food-related problematic symptoms in older adults in residential care homes.

## Abbreviations

6SQuID: 6 steps for quality intervention development
IDDSI: International Dysphagia Diet Standardization Initiative
MUST: Malnutrition Universal Screening Tool
NHS: National Health Service
